# Hysteroscopic management of uterine diverticulum after myomectomy: a case report

**DOI:** 10.1186/s12905-023-02606-7

**Published:** 2023-08-28

**Authors:** Yusuke Sako, Tetsuya Hirata

**Affiliations:** 1 Department of Obstetrics and Gynecology, St Luke’s International Hospital, 9-1 Akashi-Cho, Chuo-Ku, Tokyo, 104-8560 Japan; 2 Department of Obstetrics and Gynecology, University of Tokyo, Tokyo, Japan

**Keywords:** Uterine diverticulum, Hysteroscopic surgery, Myomectomy, Cesarean scar defect

## Abstract

**Background:**

A uterine diverticulum is defined as the presence of a niche within the inner contour of the uterine myometrial wall. Although secondary uterine diverticula can occur after hysterotomy such as cesarean section, reports of diverticula after myomectomy are extremely rare.

**Case presentation:**

A 45-year-old nulliparous woman undergoing infertility treatment was referred to our hospital because of abnormal postmenstrual bleeding after myomectomy. Transvaginal sonography and magnetic resonance imaging revealed a diverticulum in the isthmus. Fat-saturated T1 image showed a blood reservoir in the diverticulum. Hysteroscopic surgery was performed to remove the lowed edge of the defect and coagulate the hypervascularized area. Two months after surgery, the abnormal postmenstrual bleeding and chronic endometritis improved.

**Discussion and conclusions:**

This report highlights the similarities of the patient’s diverticulum to cesarean scar defects in terms of symptoms and pathophysiology. First, this patient developed a diverticulum with hypervascularity after myomectomy and persistent abnormal bleeding. Second, after hysteroscopic surgery, the symptoms of irregular bleeding disappeared. Third, endometrial glands were identified within the resected scar tissue. Fourth, preoperatively identified CD138-positive cells in endometrial tissue spontaneously disappeared after hysteroscopic resection. To the best of our knowledge, this is the first report of symptomatic improvement following hysteroscopic surgery in a patient with an iatrogenic uterine diverticulum with persistent irregular bleeding after myomectomy.

## Background

A uterine diverticulum is defined as the presence of a niche, communicating with the endometrial cavity, within the inner contour of the uterine myometrial wall. Uterine diverticula are divided into congenital and secondary [1, 2]. Secondary uterine diverticula occurs mostly after cesarean section [3]. Occasionally, healing of the hysterotomy incision leaves areas of myometrial loss of continuity, creating pouch-like defects known as an isthmocele or a cesarean scar defect (CSD) [3]. Such defects act as reservoirs that collect menstrual blood, causing postmenstrual bleeding, pelvic pain, and secondary infertility [3]. Patients with symptomatic CSD are candidates for surgical therapy and have traditionally been treated hysteroscopically, laparoscopically, or using a combination of both techniques [3, 4]. Although very rare, there have been reports of diverticula after myomectomy [5]. However, to the best of our knowledge, there have been no reports of hysteroscopic resection for iatrogenic uterine diverticulum after myomectomy. Therefore, we report a case of hysteroscopic resection of an iatrogenic uterine diverticulum after myomectomy, which had characteristics similar a CSD.

## Case presentation

**Fig. 1:**
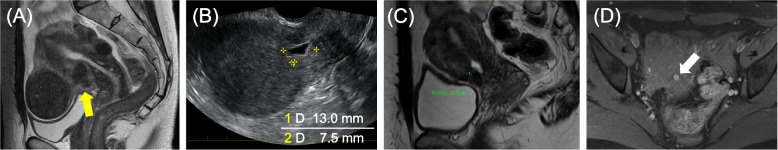
**A** Sagittal view of a T2-weighted magnetic resonance image showing multiple uterine fibroids before myomectomy. The yellow arrow denotes the uterine fibroid in the isthmus. **B** Transvaginal sonography showing a 13.0 × 7.7 mm diverticulum. **C** Sagittal view of a T2-weighted magnetic resonance image showing a residual myometrium thickness of 13.6 mm and fluid retention in the diverticulum. **D** Transverse view of a fat-saturated T1-weighted magnetic resonance image showing high signals (*white arrow*) inside the diverticulum, suggestive of blood retention

**Fig. 2:**
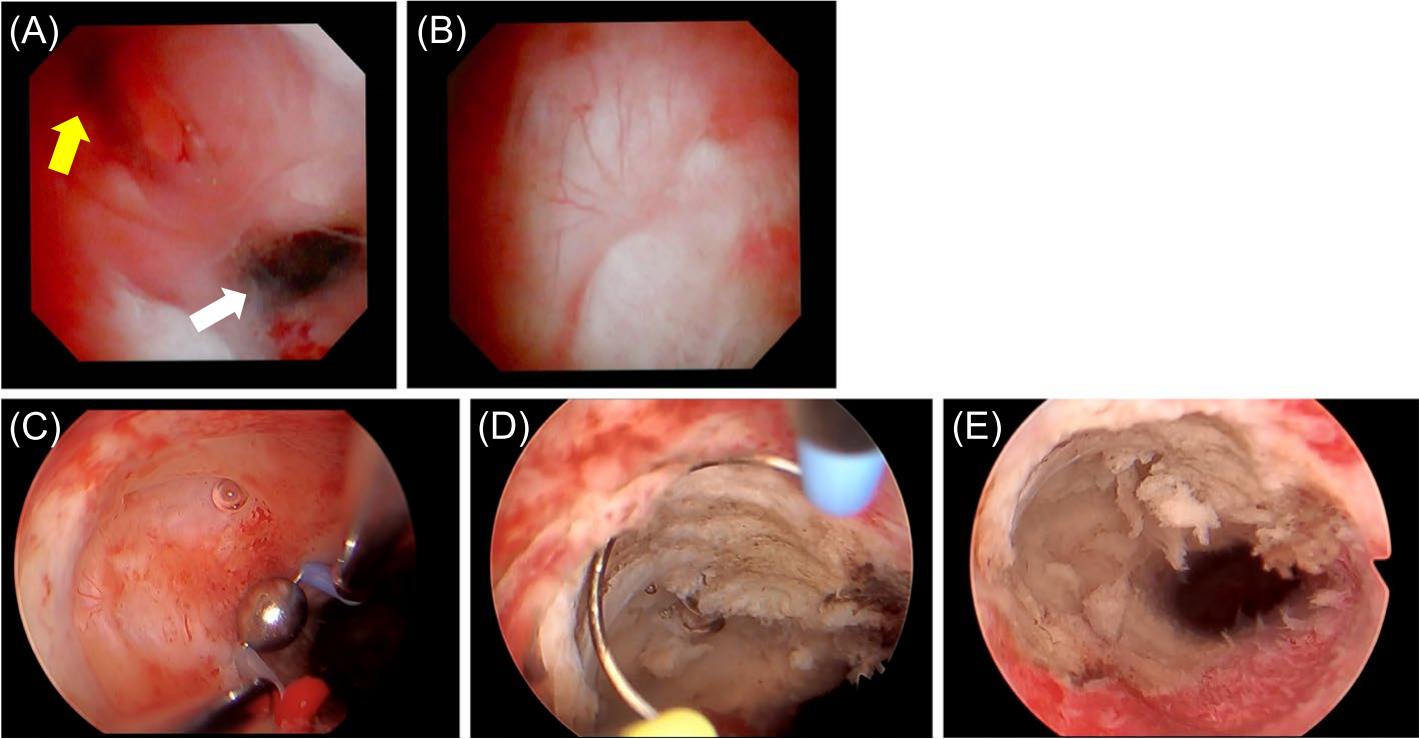
**A** Preoperative hysteroscopy revealing blood retention in the diverticulum (*yellow arrow*). The white arrow indicates the uterine cavity. **B** Preoperative hysteroscopy showing dendritic vessels consistent with the defect. **C** Operative hysteroscopy showing the bottom of the depression. **D**-**E** The inferior edge of the defect was resected and coagulated

**Fig. 3:**
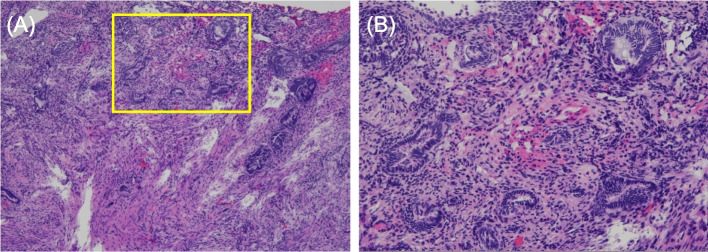
Histological examination of the surgical specimen identified endometriotic glands in lower (× 40) (**A**) or higher (× 100) (**B**) magnification. Area outlined by the yellow rectangle in (**A**) is magnified in (**B**)

**Fig. 4:**
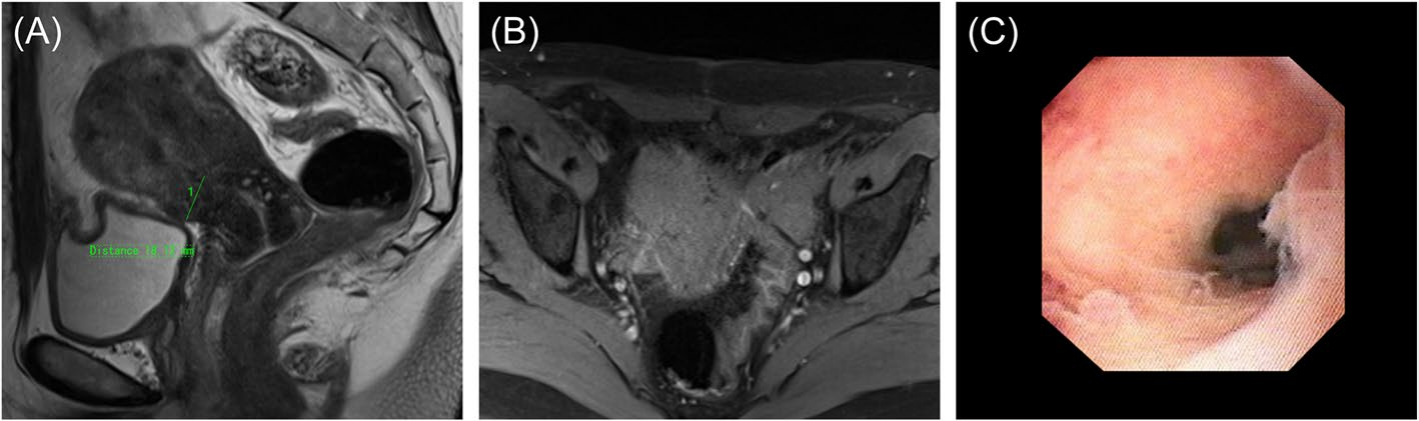
**A** Sagittal view of a T2-weighted magnetic resonance image showing a residual myometrium thickness of 18.1 mm and the disappearance of fluid retention in the diverticulum after hysteroscopic surgery. **B** Transverse view of a fat-saturated T1-weighted magnetic resonance image showing the disappearance of high-signal areas suggestive of blood. **C** Postoperative hysteroscopy revealed neither hypervascularized area nor dendritic vessels

**Fig. 5:**
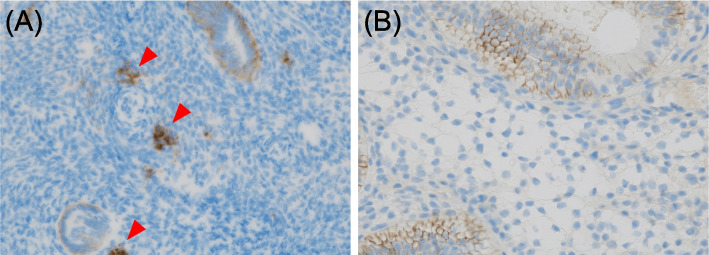
Immunohistochemical staining images for CD138 in the endometrium before (**A**) and after (**B**) surgery. The red arrow heads indicate CD138 positive plasma cells

A 45-year-old nulligravida woman with a history of laparoscopic ovarian endometrioma excision and abdominal myomectomy (27 fibroids, largest diameter 10 cm), presented to a fertility clinic. Transvaginal sonography (TVS) and magnetic resonance imaging (MRI) revealed multiple fibroid recurrences, the largest being 8 cm in diameter (Fig. 1A). Preoperatively, infertility treatment by assisted reproductive technology was administered, and three blastocysts were cryopreserved. The patient subsequently underwent a second abdominal myomectomy, in which 26 fibroids were removed. Postoperatively, she complained of prolonged menstruation (15 days) and persistent brown discharge. Six months postoperatively, TVS revealed a 13.0 × 7.5 mm-sized diverticulum in the isthmus (Fig. 1B). MRI revealed a residual myometrial thickness (RMT) of 13.6 mm (Fig. 1C), and fat-saturated T1-weighted images showed high-signal areas, indicating the presence of a blood reservoir in the same area (Fig. 1D). She was referred to our hospital because her symptoms persisted during the embryo transfer cycle. Hysteroscopy revealed blood accumulation in the uterine diverticulum and the presence of a hypervascularized area and dendritic vessels consistent with the lesion (Fig. 2A, B). We performed hysteroscopic surgery to cut the lower edge of the defect and coagulate the hypervascularized areas to prevent pooling of blood in the diverticulum (Fig. 2C–E). Hysteroscopic surgery was performed 7 days after the patient’s last menstruation. Surgical procedures were performed using a 9-mm resectoscope with 0° optical system (TCRis; Olympus Tokyo, Japan) under general anesthesia. The cervical canal was dilated to 10.5 mm using Hegar dilators, and the uterine cavity was distended at 100 mmHg pressure using physiologic saline. Histopathology of the excised specimens revealed endometrial glands within the scar tissue (Fig. 3). Two months postoperatively, the menorrhagia improved and the persistent brown discharge disappeared. In addition, postoperative MRI revealed an RMT of 18.1 mm (Fig. 4A), and fat-saturated T1-weighted images showed disappearance of the high-signal areas (Fig. 4B). Hysteroscopy also showed no hypervascularized area. (Fig. 4C). Preoperative endometrial biopsy and immunohistochemical staining using CD138 antibody showed the presence of 10 plasma cells per 20 high-power fields (HPF) of endometrial tissue (Fig. 5A), leading to the diagnosis of chronic endometritis (CE). After surgery, the CD138-positive plasma cells disappeared (Fig. 5B). The patient underwent single blastocyst transfer 3 months after surgery, and this did not result in pregnancy. She is currently awaiting her next embryo transfer.

## Discussion and conclusions

To the best of our knowledge, this is the first report of symptomatic improvement following hysteroscopic surgery of an iatrogenic uterine diverticulum with persistent irregular bleeding after myomectomy. This case of uterine diverticulum after uterine fibroid enucleation is similar to CSD in terms of symptoms and pathophysiology. First, this patient developed a symptomatic diverticulum after myomectomy. Second, the irregular bleeding resolved after hysteroscopic surgery, and the high-intensity signal indicating blood retention in the diverticulum disappeared on fat-saturated T1-weighted MRI. Third, endometrial glands were identified within the resected scar tissue. Fourth, CD138-positive cells in the endometrial tissue spontaneously disappeared after hysteroscopic resection.

In this case, a scar defect occurred at the uterine incision site after the resection of a uterine fibroid in the isthmus, which caused postmenstrual abnormal uterine bleeding (PAUB). Hysteroscopy revealed dendritic vascular proliferation in the defect and confirmed retention of blood in the defect. The accumulation of blood also caused high-intensity signals on fat-saturated T1-weighted images. These findings are characteristic of CSDs [3]. The presence of hemorrhagic hypervascularized areas and dendritic vessels in patients with symptomatic CSD suggests that the hemorrhage originates from scar areas [6]. Considering the similarity in hysteroscopic and MRI findings and the occurrence of abnormal bleeding at the hysterotomy site, the mechanism of uterine diverticulum development after myomectomy is very similar to that of CSD. However, very few cases of uterine diverticulum after myomectomy have been reported [5], despite an increase in reports of CSD [3]. Given the small number of reported cases of diverticula after myomectomy and their occurrence in the isthmus, the site of hysterotomy may determine the lesion type. In patients who undergo cesarean section, those with low incisions have been reported to have a sixfold higher incidence of large scar defects than those with high incisions [7], so a low incision site for myomectomy may also predispose to diverticulum development.

Because this patient had sufficient RMT, hysteroscopic surgery was chosen to resect the lower edge of the diverticulum and coagulate the hypervascularized scar area. Surgical options for CSD include hysteroscopic, laparoscopic, or combined surgery [3, 4, 8]. In CSD, RMT is a key consideration when choosing the surgical approach [3]. We chose hysteroscopic resection because in CSD hysteroscopy is preferred if the RMT is > 2–3.5 mm [3]. Hysteroscopic resection improved PAUB, and postoperative fat-saturated T1-weighted MRI images showed the disappearance of blood components in the uterine cavity. Although a decrease in postoperative RMT after hysteroscopic resection is a concern, in this patient, the RMT increased postoperatively from 13.6 mm to 18.1 mm. There have been previous similar reports of increased or nearly unchanged RMT after hysteroscopic surgery for CSD [3]. The symptom improvement, improved imaging findings, and increased RMT suggest that hysteroscopic resection was appropriate in this patient.

In CSD, endometrial glands are identified in in 21.1–42.9% of excised scar tissue samples [3, 9], as in our patient. Endometriotic lesions within the scar of CSD may contribute to blood retention [3]. Hysteroscopic excision and coagulation of the scarred area containing the endometrial glands may have contributed to the improvement of PAUB in our patient.

CD138-positive plasma cells were increased in the preoperative endometrium of this patient, and CE was diagnosed using histological criteria. The histological features of CE resolved spontaneously after hysteroscopic surgery. The incidence of CE, histologically diagnosed by the number of CD138-positive plasma cells, has been reported to be increased in patients with symptomatic CSD [10], and the CD138-positive plasma cell count is higher in the scars of patients with symptomatic CSD than in those with asymptomatic CSD [9]. Considering these reports and the similarity between this case and CSD cases, symptomatic uterine diverticula might be involved in the development of CE. In a recent report, patients with CE who had endometrial polyps, intrauterine adhesions, or uterine fibroma, removal by hysteroscopic surgery improved CE without antibiotic therapy [11]. Although this case report cannot be conclusive and further case investigations are required, hysteroscopic surgery of the uterine diverticulum may have contributed to the spontaneous improvement of CE in this case.

In conclusion, we report a case of iatrogenic uterine diverticulum after myomectomy, which was treated by hysteroscopic resection.

The clinical and pathological similarities between the diverticulum after myomectomy in this patient and those seen after cesarean section, suggest that this case might be attributable to hysterotomy scar defects. Further research is required to better understand the mechanism of this unique condition.

## Data Availability

The datasets used during the current study are available from the corresponding author on reasonable request.

## Abbreviations

CSD: Cesarean scar defect
TVS: Transvaginal sonography
MRI: Magnetic resonance imaging
RMT: Residual myometrial thickness
HPF: High-power fields
PAUB: Postmenstrual abnormal uterine bleeding
CE: Chronic endometritis
